# Hybrid nanostructures for electrochemical potassium storage

**DOI:** 10.1039/d1na00404b

**Published:** 2021-08-05

**Authors:** Ajay Piriya Vijaya Kumar Saroja, Benxia Li, Yang Xu

**Affiliations:** Department of Chemistry, University College London 20 Gordon Street London WC1H 0AJ UK y.xu.1@ucl.ac.uk; Department of Chemistry, College of Science, Zhejiang Sci-Tech University Hangzhou 310018 China

## Abstract

The wide availability and low cost of potassium resources have made electrochemical potassium storage a promising energy storage solution for sustainable decarbonisation. Research activities have been rapidly increasing in the last few years to investigate various potassium batteries such as K-ion batteries (KIBs), K–S batteries and K–Se batteries. The electrode materials of these battery technologies are being extensively studied to examine their suitability and performance, and the utilisation of hybrid nanostructures has undoubtedly contributed to the advancement of the performance. This review presents a timely summary of utilising hybrid nanostructures as battery electrodes to address the issues currently existing in potassium batteries *via* taking advantage of the compositional and structural diversity of hybrid nanostructures. The complex challenges in KIBs and K–S and K–Se batteries are outlined and the role of hybrid nanostructures is discussed in detail regarding the characteristics of intercalation, conversion and alloying reactions that take place to electrochemically store K in hybrid nanostructures, highlighting their multifunctionality in addressing the challenges. Finally, outlooks are given to stimulate new ideas and insights into the future development of hybrid nanostructures for electrochemical potassium storage.

## Abstract 2

This review presents the research progress in utilising hybrid nanostructures to address the challenges in K-ion, K–S and K–Se batteries, and highlights the promise of electrochemical potassium storage for next-generation energy storage technologies.

## Graphical abstract

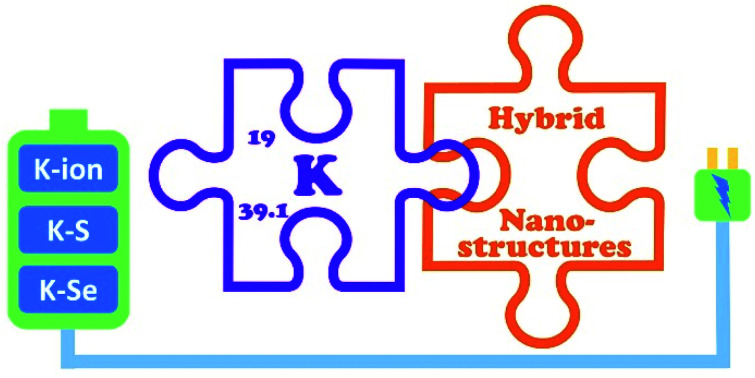

## 1. Introduction

Rechargeable batteries are sustainable energy storage technologies that support the transition to the efficient utilisation of renewable energy resources. Lithium-ion batteries (LIBs) feature high energy density and long lifespan and have dominated various sectors, ranging from portable electronics to electric vehicles.^1^ Although LIBs provide satisfactory performances in terms of energy density and durability, the availability of lithium resources in the future remains a debatable topic. The available lithium reserves are 0.0017 wt% with limited geographical locations.^2^ This imposes concerns around the long-term usage of lithium-based batteries for large scale energy storage systems and electric vehicles. The constraint in the available lithium resources urges the research community to investigate alternative battery chemistries that utilise earth-abundant elements and can provide a sustainable energy solution in the long run. Potassium has a much higher abundance (1.5 wt%) than Li, and potassium battery chemistry shares many similarities with the lithium counterpart, which provides a promising avenue for a cost-effective and electrochemically feasible battery technology.^3–5^ Besides the natural abundance, there are several benefits that favour the move from lithium batteries to potassium batteries (Table 1). First, unlike Li, K does not form alloys with Al, which makes it feasible to use Al as the current collector of both the cathode and anode^6^ in a potassium battery cell, which reduces the production cost associated with the use of a Cu current collector. Second, the lower electroplating voltage of K^+^/K (−0.15 V *vs.* Li/Li^+^) reduces the risk of potassium plating when using anode materials with a low operating potential. This indeed makes it possible to operate potassium batteries in a wide voltage window of 4.6 V (*vs.* 4.5 V for Li).^7^ Third, K ions have a lower charge density than Li ions and thus tend to form a smaller solvated ion in carbonate-based electrolytes (solvated K^+^*vs.* Li^+^: 3.6 *vs.* 4.8 Å, in propylene carbonate (PC)). This results in a high mobility and transport number of solvated K ions and benefits the rate capability of potassium batteries.^5^ Finally, potassium batteries are relatively safer when compared to lithium batteries due to a lower melting point of K (63.4 *vs.* 180.5 °C), reducing the possibility of dendrites penetrating the separator and preventing the short circuit and thermal runaway.^7,8^ Therefore, electrochemical K storage is a promising energy storage solution and as a result, has gained rapidly increasing attention in the research community.

**Table 1:** Comparison of the key features of potassium and lithium

	K	Li
Electrochemical potential^9^ (V *vs.* SHE)	−2.93	−3.01
Ionic radius (nm)	0.138	0.076
Stokes radius in PC^10^ (nm)	0.36	0.48
Ionic conductivity in PC^10^ (S cm^2^ mol^−1^)	15.2	8.3
Melting point (°C)	63.4	180.5
Electrochemical plating potential^11^ (V *vs.* Li^+^/Li)	−0.15	0
Cost^12^ (USD/ton)	1000	6500
Abundancy (wt%)	2.09	0.0017

Achieving high performance of electrochemical K storage is strongly determined by the innovation and development of electrode materials. The design of electrode materials should enable high structural stability and fast reaction kinetics and be applicable to various types of potassium batteries, including potassium-ion (KIBs), potassium–sulphur (K–S), and potassium–selenium (K–Se) batteries. The K storage process in these batteries involves intercalation,^13,14^ conversion,^15,16^ and/or alloying reactions,^17,18^ among which all three types of reactions are seen in KIBs and the conversion reaction is specifically employed in K–S and K–Se batteries. The intercalation reaction offers good durability with a reasonable storage capacity, whilst the conversion and alloying reactions deliver a high storage capacity but are limited by a poor capacity retention due to the significant volume change of electrode materials. Research on electrochemical K storage has been heavily focused on the utilisation of nanostructured electrode materials due to the intrinsic features of nanomaterials such as a large surface area, abundant active sites, and shortened K^+^ diffusion path length. Even though these features can individually or collectively contribute to enhancing the capacity and lifespan and improving the reaction kinetics of potassium batteries, when utilising a single nanomaterial, there are drawbacks caused by the features simultaneously. For instance, a high surface area could cause undesirable side reactions at the electrode–electrolyte interface, surface adsorption of K ions rather than intercalation, and low initial coulombic efficiency (ICE). In this regard, hybrid nanostructures have come into the spotlight as a highly regarded electrode structure in potassium batteries. The advantage of hybrid nanostructures lies in collectively utilising the function of each structural component and more importantly enabling a synergetic effect among the components, which solves issues (will be discussed later) that a single nanostructure has a limited ability to solve.

Given the research attention that electrochemical K storage has received, this review presents an overview of the recent advancement in applying hybrid nanostructures in potassium batteries. We emphasise on the challenges that the electrode materials of potassium batteries are facing and building on the challenges, we discuss in detail the development of hybrid nanostructures and their roles that are responsible for mitigating the challenges of the electrode materials of KIBs, K–S and K–Se batteries. Also, outlooks are given to highlight open questions that are worth being investigated to allow further development of hybrid nanostructures in electrochemical K storage. There have been a few reviews summarising the research progress of KIB electrode materials^4,14,16,19,20^ and the correlation between the electrochemical performance and dimensionality of anode materials,^8^ but to the best of our knowledge, this review is the first comprehensive summary of hybrid nanostructures for a variety of electrochemical K storage applications. We hope that this review could provide a useful platform for future studies of electrochemical K storage to build on and inspire ideas in wider scientific communities outside of battery and electrochemistry.

## 2. Challenges of electrode materials in electrochemical K storage

### 2.1 Challenges of electrode materials in KIBs

**Fig. 1:**
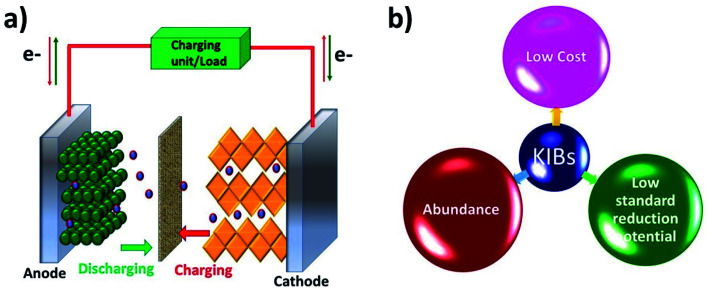
(a) Schematic of the operation of KIBs and (b) advantages of KIBs.

Like a LIB, a KIB consists of an anode, a cathode, and an electrolyte (Fig. 1). The battery works based on reversible K^+^ migration between the anode and cathode, resulting in oxidation and reduction reactions at the two electrodes. The storage capacity and energy density of the battery depend on the material design of the anode and cathode. The electrochemical mechanism of storing K^+^ in anode materials can be broadly categorised into three types, *i.e.*, intercalation, conversion and alloying reactions, where the cathode storage mechanism is predominantly based on the intercalation reaction.^19^ The following two sections will discuss the challenges that exist in the electrochemical K storage in KIB electrode materials.

#### 2.1.1 Challenges of intercalation-type electrode materials in KIBs

Graphite has been studied as a KIB anode due to the feasible K (de)intercalation that involves three stages: stage I KC_8_, stage II KC_24_ and stage III KC_36_.^21^ Each step builds on the continuous potassiation of the intercalated graphite compound from the previous stage, delivering a theoretical capacity of 279 mA h g^−1^.^22,23^ Studies have shown that graphite suffers from poor cycling stability and low ICE.^22^ Its structural instability with a capacity decay of 0.053% per cycle emerged due to its volume expansion of ∼61% upon potassiation.^21^ Two-dimensional (2D) graphene, either a monolayer or a few stacked layers, is favourable for the accommodation of volume expansion of the thin layers and extra K storage by surface storage, but the closely stacked graphene sheets could block K migration at higher current densities,^24^ resulting in unsatisfactory rate capability. Hard and soft carbons have randomly distributed graphitic domains with a large interlayer spacing and nanopores between the domains.^25^ The graphitic domains act as the active sites for K intercalation and the nanopores provide K surface adsorption sites; therefore, they exhibit a higher capacity and durability than graphite.^26,27^ Hard carbon and soft carbon are disordered carbon materials with the existence of short-range graphitic domains and long-range amorphous regions with defects and pores. Hard carbon is non-graphitisable but the degree of graphitisation of soft carbon can be tuned by increasing the temperature of graphitisation. The charge storage mechanism of hard carbon has been dominated by capacitive storage due to the poor diffusion of K^+^ below 0.4 V.^28^ Hence, the discharge profiles of hard carbon show a sloping feature in the voltage region of >1 V, where most capacity is obtained.^26^ This has a negative effect on achieving a high voltage of KIB full-cells. On the other hand, soft carbon with a semi-graphitic nature can promote K^+^ intercalation, and a high capacity can be obtained at a low operating voltage of <1 V.^29^ However, increasing the cycling stability of soft carbon remains challenging. Besides carbon, some metal oxides with an open framework/layered structure possess K intercalation sites, but the poor reaction kinetics and low electronic conductivity of metal oxides result in unsatisfactory K storage capacity and rate performance. For instance, Ti_6_O_11_ and K_2_Ti_4_O_9_ exhibited a capacity less than 200 mA h g^−1^ and the capacity declined quickly at a current density greater than 3 A g^−1^.^30,31^ Although Nb_2_O_5_ exhibited better rate performance due to its pseudocapacitive effect, the capacity was observed to be low.^32^

#### 2.1.2 Challenges of conversion- and alloying-type electrode materials in KIBs

Conversion- and alloying-type KIB electrode materials have the advantage of a high theoretical capacity over intercalation-type materials, which is due to the multi-electron transfer occurring in the reactions. A conversion reaction is often seen in various KIB anodes such as metal oxides, sulphides, selenides, and phosphides. The reaction results in the reduction of the anodes to a metal and the corresponding K compound. Besides the high theoretical capacity, the phase formation of conversion-type electrode materials can be fine-tuned due to their rich crystal structures and electrochemistry towards K.^33–35^ They are also generally considered to be safer than carbons.^7^ However, they are facing the challenges of low electronic and ionic conductivity as well as complicated phase change during K storage, which could lead to a large volume change, causing the pulverisation and delamination of the materials and even KIB cell failure.^36^ It is worth pointing out that the conversion reaction of metal sulphides and selenides can cause the dissolution of K polysulphides/polyselenides in the electrolyte and trigger the shuttle effect,^15,37–39^ which results in rapid capacity fade and irreversible side reactions in the KIB cell.

Alloying-type KIB electrode materials refer to the elements that can electrochemically alloy with K, such as group XIV (Si, Ge, Sn and Pb) and XV elements (P, Sb and Bi). An alloying reaction can deliver a very high theoretical capacity due to the more electrons transferred compared to intercalation- and conversion-type electrode materials. For instance, Ge and P form stable alloys GeK_3_ and KP with K^+^, respectively, enabling theoretical capacities of 1108 and 843 mA h g^−1^.^40,41^ The major shortcoming of alloying-type materials is the structural instability caused by the huge volume expansion during K insertion.^42^ This leads to the pulverisation of the materials and poor cycling stability. In the case of electrochemically storing K^+^ in Sn, the formation of the intermediate phase K_4_Sn_9_ and final phase KSn led to a volume change of 113% and 197%, respectively.^18,43^ Similar to Sn, the electrochemical reaction between Bi and K^+^ to form K_3_Bi led to a volume change of ∼406%.^44^ The accompanied volume strain can cause the structural disintegration and agglomeration of the materials, and subsequently they can detach from the current collector, leading to an increase in the internal resistance of the cell and poor cycling stability.^45,46^ More critically, irreversible reactions could be intensified with increasing freshly formed material surface over the cycles, resulting in a low ICE^47,48^ and a slow CE increase.^49^

### 2.2 Challenges of electrode materials in K–S and K–Se batteries

**Fig. 2:**
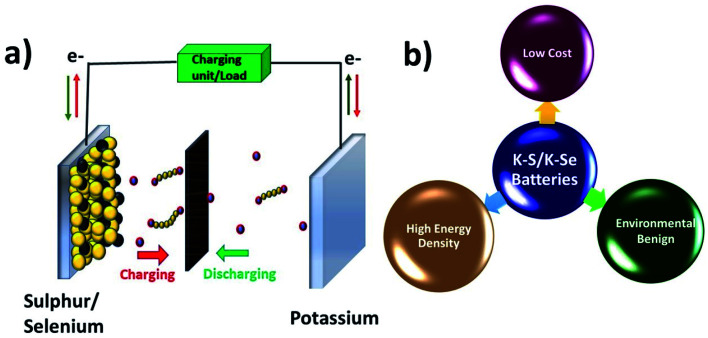
(a) Schematic of the operation of K–S batteries and (b) advantages of K–S/K–Se batteries.

K–S and K–Se batteries are two emerging battery technologies that have great potential for cost-effective and large-scale energy storage applications. Due to the high similarity between K–S and K–Se batteries, they are discussed together in this section. A typical battery cell consists of a metallic K anode, a S or Se cathode, and a separator sandwiched between the two electrodes and soaked in an organic electrolyte (Fig. 2a). Battery chemistry is based on the conversion reaction between K and S(Se) on the cathode side and the plating/stripping of K on the anode side. During a discharge process, K^+^ is electrochemically stripped from the metallic K and S(Se) is reduced to K_2_S (K_2_Se) as the end-discharge product through the formation of various intermediate polysulphides S_*n*_^2−^ (polyselenide Se_*n*_^2−^, 2 < *n* < 8). The use of S and Se cathodes is the key to realising a high energy density of K–S and K–Se batteries, which could be in general 2–5 times higher than that of KIBs. The two-electron redox process is expected to deliver a theoretical capacity of 1675 mA h g^−1^ and a gravimetric energy density of 1023 W h kg^−1^ in K–S batteries,^50^ considering an average discharge voltage of 2.1 V. The numbers are significantly low in the case of K–Se batteries, 675 mA h g^−1^ and 1.8 V,^51^ but Se has a comparable volumetric capacity to S (3250 *vs.* 3470 mA h cm^−3^) due to its higher density (4.8 g cm^−3^),^52^ which is desirable for modern battery technologies dealing with a limited battery packing space. In addition, the use of S and Se can greatly cut down the cost of battery production due to the absence of transition metals such as Co and Ni commonly seen in LIB production.

Although K–S and K–Se batteries have several benefits (Fig. 2b), they are facing critical challenges before they could fully deliver on the promise. The first and foremost challenge is the shuttle effect (also seen in sulphides and selenides used as KIB electrode materials) that is originated from the dissolution of the discharge intermediates (S_*n*_^2−^ and Se_*n*_^2−^) into the electrolyte and refers to the migration of the intermediates to the K anode. The shuttle effect results in the deposition of the intermediates on K and the loss of active cathode material over the course of cycles. Second, the volume expansion of S and Se occurs during the conversion reaction and leads to the structural deformation of S and Se, having negative influence on the CE, self-discharge rate, and durability of the battery cell. Third, the phase transition from high order intermediates (4 < *n* < 8) to low order ones (2 < *n* < 4) has poor kinetics, which limits the full use of the cathode and its rate capability. Last but not the lease, the insulating nature of S adds another challenge to K–S batteries, for which a conductive carbon component (will be discussed in Section 4.1) is needed. Se has a much higher electronic conductivity (Se *vs.* S: 1 × 10^−3^*vs.* 5 × 10^−28^ S m^−1^),^51^ which might offer an extra advantage to K–Se batteries.

**Fig. 3:**
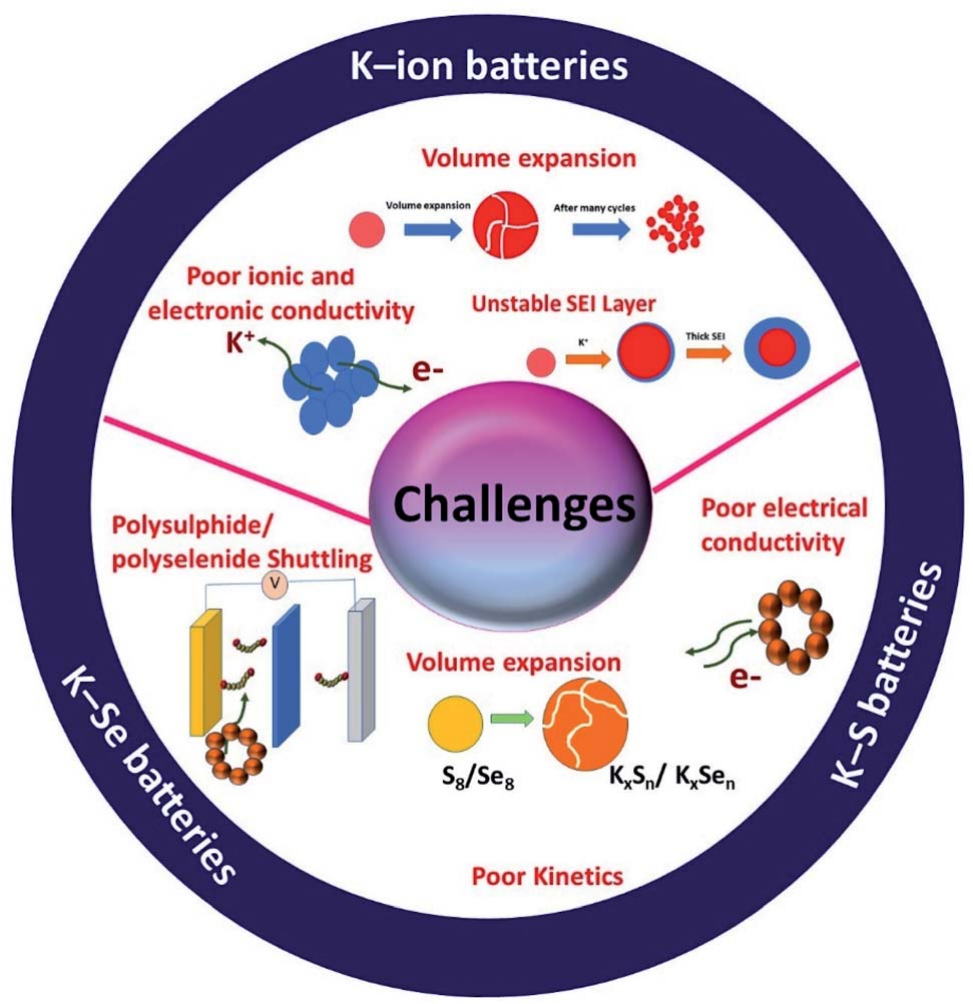
Schematic of the challenges of potassium batteries.

In summary, there are unique features in KIBs, K–S and K–Se batteries but they also are facing unique challenges that are associated with the individual battery chemistry and the corresponding electrochemical K storage process (Fig. 3). Hybrid nanostructures have a high compositional and structural adjustability and when used as the electrodes of potassium batteries, the adjustability allows hybrid nanostructures to be purposely constructed, targeted at a specific challenge(s). The great promise of hybrid nanostructures has been solidified by the advancement of electrochemical K storage.

## 3. Hybrid nanostructures for KIBs

### 3.1 Carbon/carbon hybrid nanostructures

**Fig. 4:**
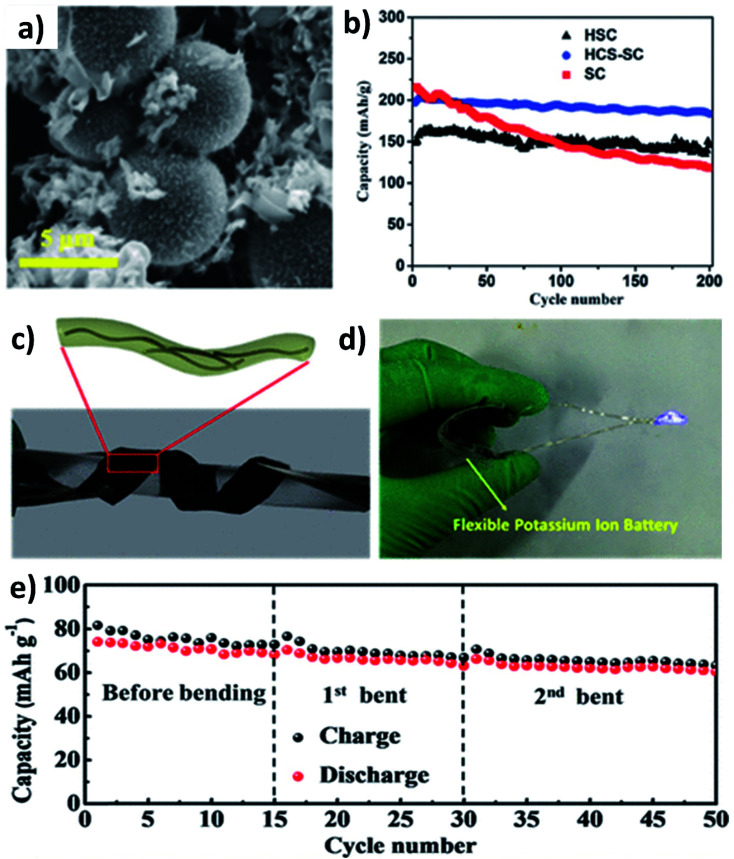
(a) SEM image of the HCS–SC hybrid and (b) cycling performance of HCS, SC and HCS–SC. Reproduced with permission. Copyright 2017. Wiley-VCH. (c) and (d) Schematic representation and photographic image of the CF/CNT hybrid flexible electrode and (e) electrochemical performance under bending conditions of a flexible KIB fabricated using the CF/CNT hybrid. Reproduced with permission. Copyright 2019, American Chemical Society.

As previously discussed, hard carbon has good cycle stability but exhibits poor rate capability, whilst soft carbon can store K^+^ at high rates but have unsatisfactory stability; hence, hybridising hard carbon and soft carbon can integrate their individual benefits to obtain good rate performance and durability simultaneously. Jian *et al.* hybridised 20% soft carbon in a hard carbon matrix (HCS–SC) using the ball milling method and compared the performance of HCS and SC with that of HCS–SC to illustrate the significance of using the hybrid structure.^53^ SC with a size of 50 nm was anchored on the surface and in the pores of HCS with a micrometre size (Fig. 4a) and the drastic decrease of the surface area of HCS from 175 to 20 m^2^ g^−1^ indicated the successful hybridisation. Although the ICE was in the order of HCS > HCS–SC > SC (76% > 63% > 67%) due to the K^+^ trapping in SC, the rate performance of HCS–SC was significantly improved from 45 to 81 mA h g^−1^ at 10C compared to HCS, which was ascribed to the higher electronic conductivity of SC over HCS (5.7 *vs.* 1.7 S m^−1^). HCS–SC showed no significant change in capacity when cycling at 0.1C compared to HCS and SC, and it exhibited an excellent capacity retention of 93% at 1C after 200 cycles (Fig. 4b). Enhancing surface storage is an alternative approach to enhance the rate capability of KIB electrode materials. To this end, carbon materials such as graphene, carbon nanospheres, carbon fibres (CFs), and carbon nanotubes (CNTs) have a large surface area and are suitable to form hybrid materials with high surface charge storage. Wei's group designed a hybrid nanostructure of CF@CNT using the electrospinning technique followed by carbonisation.^54^ CF@CNT had a flexible structure enabled by a three-dimensional (3D) interconnected framework with CNTs dispersed in CFs (Fig. 4c and d), making the hybrid a freestanding electrode. It exhibited the synergistic feature of the surface charge storage offered by CFs and the good electrical conductivity and mechanical stability offered by CNTs. As a result, CF@CNT delivered a reversible specific capacity of 108 mA h g^−1^ at 5C with a capacity retention of 98% at the end of 300 cycles. The structural stability of the freestanding anode was demonstrated by the consistent electrochemical performance throughout the continuous bending actions tested in a KIB full-cell (Fig. 4e).

### 3.2 Metal oxide/carbon hybrid nanostructures

In this section, we will discuss the advancement of utilising metal oxide hybrid nanostructures with focus on the K storage mechanism of electrode materials, *i.e.*, intercalation, conversion, and coupled conversion–alloying reactions. The design of metal oxide hybrid nanostructures can improve reaction kinetics *via* shortening the K^+^ diffusion path, enhancing electronic conductivity, alleviating volume expansion, and preventing the pulverisation of electrode materials.

**Fig. 5:**
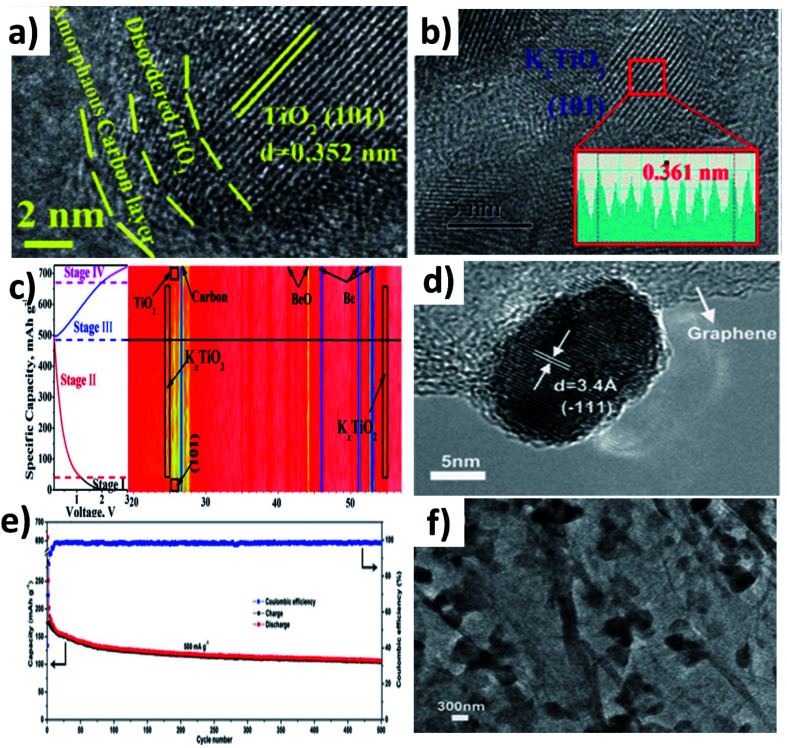
(a) HRTEM image of H-TiO_2_@C, (b) HRTEM image of H-TiO_2_@C cycled for 100 cycles in the discharged state, and (c) *in situ* XRD of H-TiO_2_@C in different charge and discharge states. Reproduced with permission. Copyright 2019, Elsevier. (d) Cycling performance of MoO_2_/rGO at 500 mA g^−1^, (e) HRTEM image of MoO_2_/rGO, and (f) TEM image of MoO_2_/rGO after 500 cycles. Reproduced with permission. Copyright 2018, Wiley-VCH.

TiO_2_ is an intercalation-type KIB anode, and it suffers from poor conductivity and reaction kinetics, showing inferior rate capability. Li *et al.* synthesised a hybrid nanostructure comprising disordered TiO_2_ sandwiched between crystalline TiO_2_ and carbon (H-TiO_2_@C) (Fig. 5a).^55^ The disordered TiO_2_ layer was formed as a result of the hydrogenation process and aimed to establish a stable interface between crystalline TiO_2_ and C. The hybrid simultaneously realised good reaction kinetics, structural stability to control volume expansion, and the elimination of by-product aggregation. It exhibited pseudocapacitive K storage at a voltage above 1.2 V and K intercalation below 1.2 V to form K_*x*_TiO_2_, and the reversibility of the K storage process was confirmed by micro-structural and phase characterisation (Fig. 5b and c). Benefiting from the strong interaction between TiO_2_ and carbon, H-TiO_2_@C exhibited superior cycling stability over 1200 cycles, and the K^+^ diffusion coefficient (*D*_K_) was 8 times higher in H-TiO_2_@C (7.32 × 10^−14^ cm^−2^ S^−1^) than in TiO_2_ (0.89 × 10^−14^ cm^−2^ S^−1^) due to the low energy barrier for K migration. The considerable volume change that occurs in layer-structured MoO_2_ during K (de)intercalation has been seen as a downside of MoO_2_ as an anode material. Liu *et al.* prepared a hybrid nanostructure consisting of MoO_2_ microspheres with a size of 150–320 nm and reduced graphene oxide (rGO) (Fig. 5d).^56^ Hybridising MoO_2_ with rGO increased the specific area to 82.55 m^2^ g^−1^ from 17.56 m^2^ g^−1^ along with an increase in pore volume. The high specific surface area of the hybrid structure provided more active sites for K storage and the increase in pore volume aids in accommodating volume change. As a result, the hybrid structure exhibited a 1.3-fold increase in reversible capacity compared to MoO_2_ and a stable cycling life for 500 cycles (Fig. 5e). The morphology of the hybrid structure was retained after cycling, suggesting its structural stability (Fig. 5f).

**Fig. 6:**
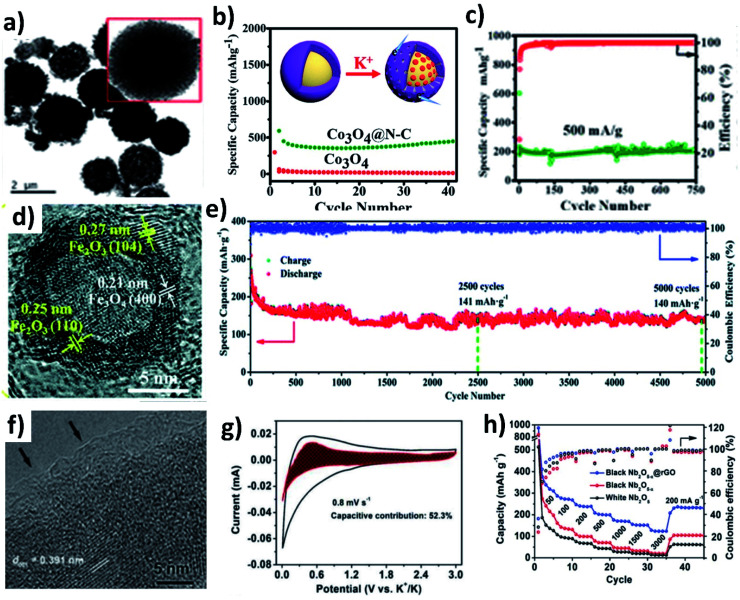
(a) TEM image of Co_3_O_4_@NC, (b) cycling stability of Co_3_O_4_ and Co_3_O_4_@NC at 50 mA g^−1^, and (c) cycling stability of Co_3_O_4_@NC at 500 mA g^−1^. Reproduced with permission. Copyright 2020, American Chemical Society. (d) HRTEM image and (e) cycling stability of Fe_*x*_O@NG. Reproduced with permission. Copyright 2018, Royal Society of Chemistry. (f) HRTEM image of Nb_2_O_5−*x*_@rGO, (g) CV curves of Nb_2_O_5−*x*_@rGO at 0.8 mV s^−1^ with the shaded region representing surface capacitive current, and (h) rate performance of Nb_2_O_5−*x*_@rGO. Reproduced with permission. Copyright 2019, Wiley-VCH.

The conversion reaction kinetics and the associated volume change of metal oxides can be improved by (i) providing a conductive framework, (ii) introducing pseudocapacitive charge storage, (iii) nano-structuring metal oxides, (iv) introducing a buffer layer to minimise mechanical strain, and (v) designing hollow structures to accommodate the volume change and facilitate electrolyte penetration. The design of hybrid structures of conversion-type metal oxides with the integration of several inherent properties can overcome the existing drawbacks. Co_3_O_4_ is a high-capacity conversion-type KIB anode, but the experimentally available capacity has been lower than the theoretical value, due to the change in volume and poor electronic conductivity. The problem can be solved by introducing a highly conductive carbon layer over Co_3_O_4_. Adekoya *et al.* designed Co_3_O_4_ coated with nitrogen doped carbon (Co_3_O_4_@NC) using the solvothermal method.^57^ The Co_3_O_4_@NC microspheres had a uniform coating layer of NC (Fig. 6a). The hybrid nanostructure exhibited a capacity of 448 mA h g^−1^ at 50 mA g^−1^ in contrast to 10 mA h g^−1^ delivered by Co_3_O_4_ (Fig. 6b). The Co_3_O_4_ cell failed to operate at high current densities but the hybrid structure delivered ∼120 mA h g^−1^ at 2C, owing to the capacity contribution from diffusion and pseudocapacitive processes (∼67%). Moreover, the long-term stability over 750 cycles was retained for the Co_3_O_4_@NC structure due to the mesoporous nature of the hybrid structure assisting in durability (Fig. 6c). The same approach has also been applied to Fe_2_O_3_, where hollow Fe_2_O_3_ was anchored onto nitrogen doped few layered graphene to form an Fe_*x*_O@NG hybrid nanostructure with both micro- and meso-pores (Fig. 6d).^58^ The hollow interior of Fe_*x*_O can buffer the volume expansion during K storage and the interconnected graphene network can provide a conductive framework for the hybrid structure. Fe_*x*_O@NG showed stable electrochemical reversibility and excellent cycling stability by retaining 79.5% capacity over a long term of 5000 cycles (Fig. 6e). Nb_2_O_5_ is a suitable host to store large-sized Na^+^ and K^+^ owing to its large (002) *d*-spacing of 3.9 Å.^59^ The slow reaction kinetics of Nb_2_O_5_ was enhanced by introducing oxygen vacancies into the hybrid structure of Nb_2_O_5−*x*_@rGO *via* a heat treatment with NaBH_4_. An amorphous layer rich in oxygen vacancies was formed on Nb_2_O_5−*x*_ (Fig. 6f) and improved its electronic conductivity, which was proven by an enhancement of carrier density by an order of magnitude (1 × 10^19^ cm^−3^). Moreover, the presence of rGO increased the pseudocapacitive contribution (∼52%) to the K storage (Fig. 6g). As a result, Nb_2_O_5−*x*_@rGO delivered about 311 mA h g^−1^ at 50 mA g^−1^, a long-term cycling stability over 3500 cycles, and a high-rate capacity of 123 mA h g^−1^ at 3 A g^−1^ (Fig. 6f).

**Fig. 7:**
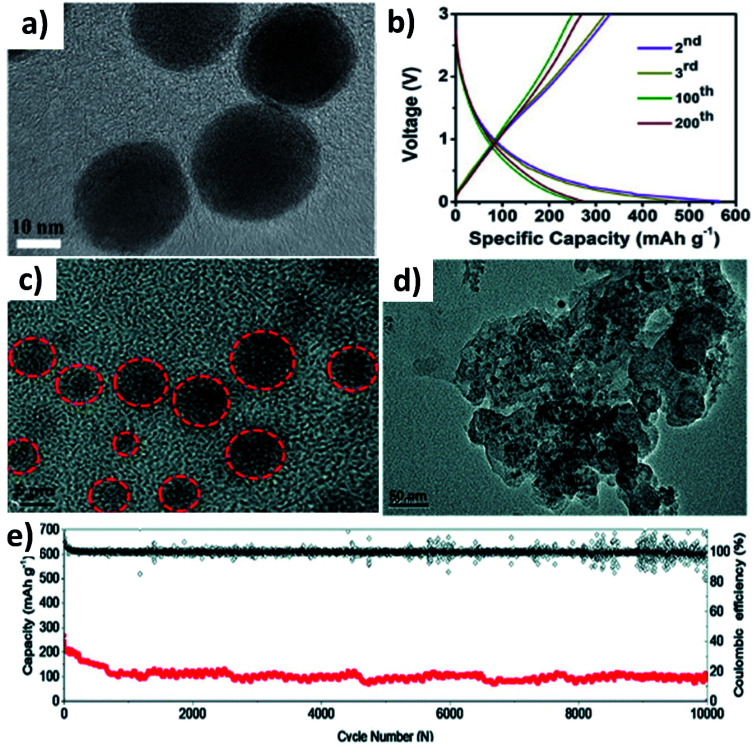
(a) TEM image and (b) charge–discharge profiles of SnO_2_@3DPC at 100 mA g^−1^. Reproduced with permission. Copyright 2019, Elsevier. (c) HRTEM image of SnO_2_@PC, (d) TEM image of SnO_2_@PC after cycling, and (e) cycling stability of SnO_2_@PC. Reproduced with permission. Copyright 2020, Royal Society of Chemistry.

Huge volume expansion is the major issue for metal oxides undergoing coupled conversion–alloying reactions to store K^+^. For instance, SnO_2_ experiences a volume expansion of ∼200% during the conversion reaction to form Sn and the subsequent alloying reaction to form K–Sn. Additionally, the agglomeration of metal oxide nanoparticles can reduce the utilisation of the oxide and even cause structural deformation due to the uneven reaction kinetics within the entire electrode. Nano-structuring SnO_2_ with a suitable carbon component can enhance the durability of SnO_2_. It has been reported that SnO_2_ nanoparticles with a size of 20–40 nm anchored on 3D porous carbon (SnO_2_@3DPC) can effectively control the volume expansion (Fig. 7a).^60^ The presence of the 3D porous carbon network provided structural integrity and effective transport of K^+^ and electrons in the hybrid nanostructure. At the same time, nanosized K_2_O formed during the conversion reaction can act as a buffer layer to accommodate volume expansion. SnO_2_@3DPC delivered a reversible capacity of 323 mA h g^−1^ at 100 mA g^−1^ (Fig. 7b) and retained ∼66% capacity after 2000 cycles at 1 A g^−1^. Further reduction in the SnO_2_ particle size down to 10 nm was found to enhance reaction kinetics and alleviate volume expansion to a greater extent. SnO_2_ nanoparticles supported on porous carbon (SnO_2_@PC) exhibited excellent performance due to the synergistic benefit of the hybrid nanostructure,^61^ where SnO_2_ particles with a unform size of 2 to 6 nm were embedded into the porous carbon without any agglomeration (Fig. 7c). Post-cycling characterisation showed no aggregation and structural deformation of the SnO_2_ nanoparticles (Fig. 7d), owing to the homogeneous distribution of the nanoparticles in the carbon matrix and its role in improving charge transfer during the coupled conversion–alloying reactions. SnO_2_@PC showed excellent stability over 10 000 cycles at 1 A g^−1^, which was the best result for KIBs when the work was published (Fig. 7e).

### 3.3 Metal chalcogenide-based hybrid nanostructures

The majority of metal chalcogenides that have been studied as KIB electrode materials include metal sulphides and selenides. They have been receiving intensive research attention due to their enhanced reaction kinetics enabled by a large interlayer spacing (for layered structures) and high ionic conductivity of the end-discharge product. We will discuss in this section the benefits of hybrid nanostructures in response to the major challenges of utilising metal chalcogenides in KIBs, *i.e.*, polysulphide/polyselenide dissolution and volume change. The former is responsible for irreversible side reactions and the latter can be found in metal oxide electrode materials too. Note that incorporating a carbon component to form a hybrid nanostructure is an effective approach to control volume expansion and has been widely studied, as exemplified in Section 3.2, and hence, we will include hybrid nanostructures with a non-carbon component, which are worth mentioning in this section.

**Fig. 8:**
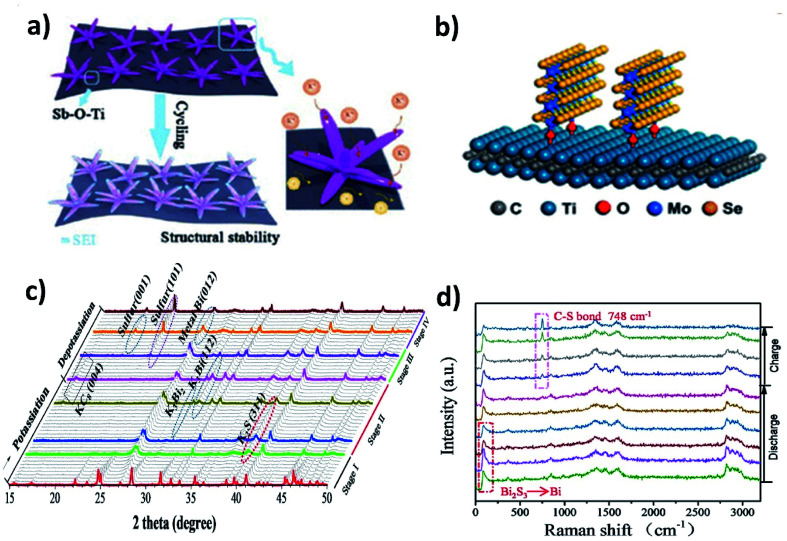
(a) Schematic illustration of the superior stability of Sb_2_S_3_@MXene during cycling. Reproduced with permission. Copyright 2020, American Chemical Society. (b) Schematic illustration of the bond formation between the MXene and MoSe_2_. Reproduced with permission. Copyright 2019, American Chemical Society. (c) *In situ* operando synchrotron X-ray diffraction and (d) *in situ* Raman spectra of Bi_2_S_3_@NG in the initial cycle. Reproduced with permission. Copyright 2020, Elsevier.

MXenes with a metallic nature and surface functional groups act as suitable substrates for anchoring metal chalcogenides and buffering volume change. Wang *et al.* used Ti_3_C_2_ to control the structural instability of Sb_2_S_3_.^62^ The hybrid nanostructure of Sb_2_S_3_@Mxene prepared by the solvothermal method formed Sb_2_S_3_ nanoflowers of about 500 nm distributed over the surface of the MXene. The hybrid showed a cycling stability over 500 cycles with a minimal capacity decay of 0.041% per cycle, whilst Sb_2_S_3_ retained only 10% after 100 cycles. The strong interaction between the MXene and Sb_2_S_3_ due to the formation of a Sb–O–Ti bond resulted in the structural stability of the hybrid (Fig. 8a). Enhancing the structural stability using MXenes can be extended to metal selenides. Huang *et al.* hybridised MoSe_2_ with Ti_3_AlC_2_ and carbon (MoSe_2_@Ti_3_AlC_2_–C) using a hydrothermal method.^63^ The vertically grown sheets of MoSe_2_ formed a strong bonding with Ti_3_AlC_2_ through surface functional groups, which prevented the aggregation of MoSe_2_ and stabilised the hybrid structure (Fig. 8b). With only 6.2 wt% loading of Ti_3_AlC_2_ in the hybrid, the capacity retention was as high as ∼98.7% after 300 cycles. Due to the weak metal–S bond, the conversion reaction of metal sulphides with K^+^ turns sulphur to polysulphides and subsequently leads to the shuttle effect. Chemical confinement is found to be effective in preventing the effect. Chen's group reduced the effect by embedding 5 nm SnS_2_ nanoparticles in a nitrogen doped graphene matrix (SnS_2_@NGO).^64^ The formation of polysulphides (K_2_S_5_) during the potassiation with SnS_2_ was confirmed by the *in situ* XRD results and the visual colour change of the separators from cycled electrodes. However, the cycling stability of SnS_2_@NGO was enhanced with the delivery of 86% of the theoretical capacity of SnS_2_ for 100 cycles. The authors concluded that the interaction between N in the hybrid structure and polysulphides was responsible for the great durability of the hybrid nanostructure. Similarly, Shen *et al.* designed a hybrid structure of Bi_2_S_3_ nanorods in nitrogen doped graphene (Bi_2_S_3_@NG)^65^ and found that severe capacity fading occurred in Bi_2_S_3_ but the bonding between Bi_2_S_3_ and NG resulted in a 4.4-fold enhancement of durability. The conversion reaction of Bi_2_S_3_ with K^+^ resulted in the formation of metallic Bi and K_2_S during discharging, but the reverse process was observed to be partially reversible due to the presence of metallic Bi and S at the end of charging (Fig. 8c). S was immobilised by the formation of a C–S bond, due to the interaction of S with NG in the hybrid, which progressed over the charging process, as confirmed by the increase in the intensity of the Raman peak (748 cm^−1^) (Fig. 8d). The hybrid Bi_2_S_3_@NG captures polysulphides through the reversible formation of C–S bonds which is solely responsible for better performance.

### 3.4 Multicomponent hybrid nanostructures

Multicomponent hybrid materials, which comprise two or more nanomaterials that have distinct characteristics, can deliver the benefit of each individual component, and more importantly realise the synergetic benefit of coupling two or three components. This section will elaborate on the following features of multicomponent hybrid nanostructures for enhancing the durability of conversion/alloying materials: (i) enhancing the structural robustness of the hybrid by limiting the volume change and agglomeration of active component(s) by the second and third components; (ii) accelerating the reversible formation of discharge by-products and reaction kinetics of active components by the second component and buffering the volume stress by the third component.

**Fig. 9:**
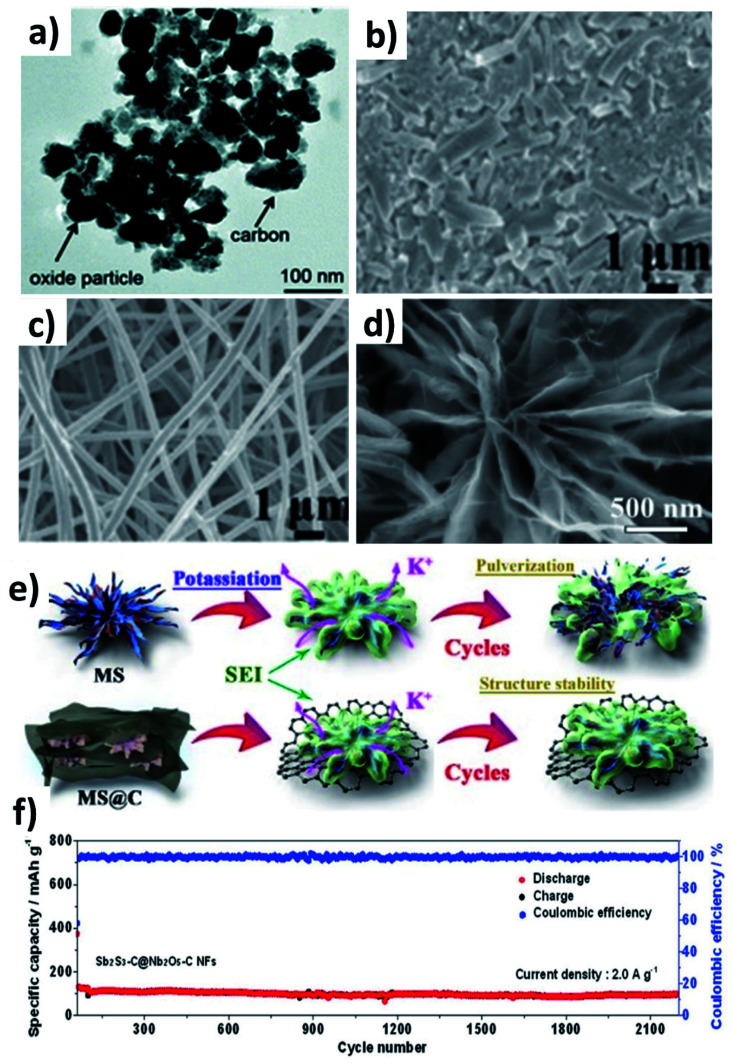
(a) TEM image of Co_3_O_4_–Fe_2_O_3_/C. Reproduced with permission. Copyright 2017, Royal Society of Chemistry. SEM images of (b) NCNF and (c) CuO–Cu/NCNF after 100 cycles. Reproduced with permission. Copyright 2020, Elsevier. (d) SEM image of MoS_2_/Sb@NG and (e) schematic illustration of controlling the volume expansion in MoS_2_/Sb@N. Reproduced with permission. Copyright 2020, Elsevier. (e) Elemental mapping and (f) cycling stability of Sb_2_S_3_–C@Nb_2_O_5_–CNF. Reproduced with permission. Copyright 2021, Wiley-VCH.

Chen *et al.* prepared Co_3_O_4_ and Fe_2_O_3_ nanoparticles (10–40 nm) embedded in super P (Co_3_O_4_–Fe_2_O_3_@SP, Fig. 9a),^66^ and the hybrid nanostructure was proven to be a high-capacity and durable anode due to the synergistic capacity contribution from Co_3_O_4_ and Fe_2_O_3_ and the reduced volumetric stress and agglomeration enabled by Fe_2_O_3_ and super P. Co_3_O_4_–Fe_2_O_3_@SP exhibited a capacity of 220 mA h g^−1^ after 50 cycles, completely outperforming Fe_2_O_3_, Co_2_O_3_ and Co_3_O_4_–Fe_2_O_3_ structures. A hierarchical CuO/Cu/nitrogen-doped carbon fibre (CuO/Cu/NCNF) hybrid took advantage of the conductive Cu to enhance the electrical conductivity and mechanical stability of the nanostructure, as well as increasing *D*_K_ (CuO/Cu/NCNF *vs.* NCNF: 5.4 × 10^−13^*vs.* 2.3 × 10^−13^ cm^2^ s^−1^).^67^ The structure of CuO/Cu/NCNF was found to be more stable after cycling compared to NCNF, as the former kept a smooth surface while the latter showed many disintegrates (Fig. 9b and c). A Sb–MoS_2_/N-doped graphene (NG) hybrid nanostructure prepared by a hydrothermal method displayed a chrysanthemum-like morphology with interconnected nanosheets (Fig. 9d).^68^ MoS_2_ with a 2D structure acted as a medium to control volume expansion and prevent the aggregation of Sb nanoparticles with NG. The hybrid not only delivered a high capacity of 350 mA h g^−1^ due to the conversion reaction of MoS_2_ and the alloying reaction of Sb and K, but also exhibited a long-term cycling durability over 1000 cycles due to the strong interaction of Sb with the NG matrix (Fig. 9e). Sb_2_S_3_ undergoes coupled conversion and alloying reactions to store K^+^ but experiences huge volume variation and the dissolution of polysulphide species during cycling; hence, Nb_2_O_5_ was used to effectively adsorb polysulphides and carbon nanofibres were used to alleviate the volume stress. The resulting Sb_2_S_3_–Nb_2_O_5_/CNF hybrid nanostructure consisted of a Sb_2_S_3_ core layer and a Nb_2_O_5_/CNF shell layer, where the shell prevents the core from coming into direct contact with the electrolyte and buffers the volume change of the core and kinetically converts the formed polysulphide species. The hybrid nanostructure exhibited a great cyclability of more than 2100 cycles at 2 A g^−1^ (Fig. 9f). The same idea of using Nb_2_O_5_/NCNF was also proven to be effective in the case of SnS_2_.^69^

### 3.5 Alloying-based metal hybrid nanostructures

The alloying reaction between an electrode material and K leads to the formation of various intermediate phases, which in turn results in the large volume change and pulverisation of the electrode. It was reported that Sn and Bi undergo a volume change of 197% and 406%, respectively, when alloying with K to form KSn and K_3_Bi.^43,70,71^ The same issue was seen for Sb,^72^ P^73,74^ and Ge^40^ as well. This section will discuss the utilisation of hybrid nanostructures to address the issue by combining nano-structuring active materials to minimise volume strain and improve ionic conductivity and hybridising with carbons to cushion volumetric strain and prevent particle agglomeration. It is worth pointing out that hybridising intermetallic compounds with a non-carbon material was demonstrated to be an interesting approach to reduce the volume change.

**Fig. 10:**
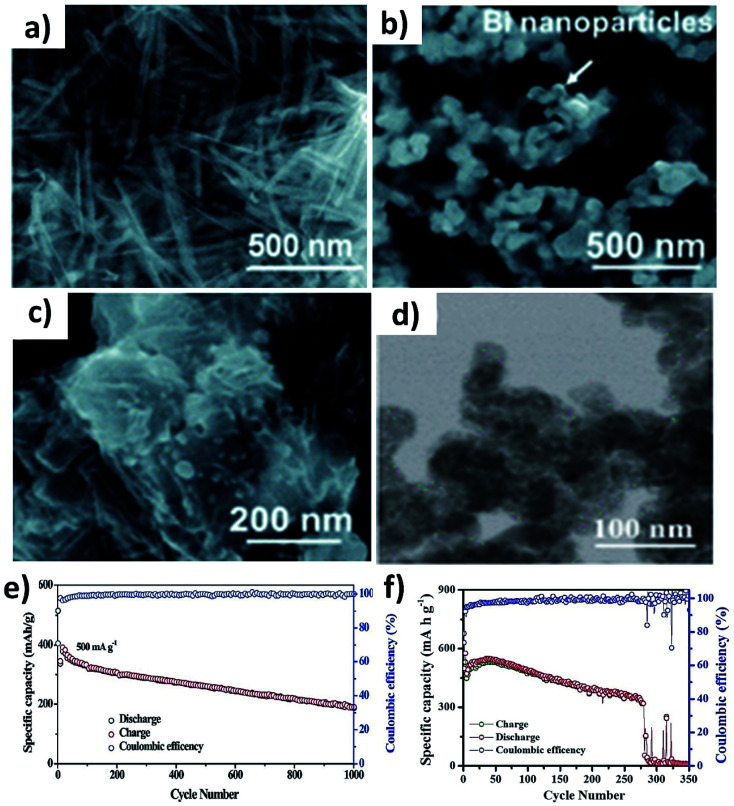
SEM images of (a) pristine Bi@NSC, (b) Bi particles and (c) Bi@NSC after 100 cycles. Reproduced with permission. Copyright 2020, Royal Society of Chemistry. (d) TEM image of Bi_0.5_Sb_0.5_@P, (e) cycling stability of Bi_0.5_Sb_0.5_@P after 1000 cycles at 500 mA g^−1^, and (f) cycling stability of ball milled Bi_0.5_Sb_0.5_@P. Reproduced with permission. Copyright 2020, American Chemical Society.

Various Bi nanostructures such as nanoparticles,^70^ nanorods,^75^ and nanosheets^76^ have been synthesised and hybridised with carbons. As the nano-structuring of Bi can minimise the strain during the volume change to a certain extent, cycling stability was limited to less than 300 cycles due to the agglomeration of the particles over the cycles.^71,76^ Zhang *et al.* designed a hybrid nanostructure of Bi nanorods and N, S co-doped carbon (Bi@NSC).^75^ The Bi nanorods with a length of 100 nm and a diameter of 20–40 nm were distributed to form a connecting network in the carbon matrix (Fig. 10a). Pure Bi powder showed a capacity of 400 mA h g^−1^ at 0.5 A g^−1^, but it drastically dropped down to zero at a rate of over 4 A g^−1^. In contrast, Bi@NSC showed a capacity of 338 mA h g^−1^ and retained 289 mA h g^−1^ at 6 A g^−1^. The NSC matrix enhanced the electronic conductivity of the hybrid, which was the key to delivering better performance at a high rate. In addition, the Bi@NSC hybrid nanostructure exhibited a capacity retention of 91% after 1000 cycles, which was much higher than that of Bi powder (61%). The agglomeration of Bi particles was not found in Bi@NSC after cycling, whilst it can be seen in the case of Bi powder (Fig. 10b and c). The KIB full-cell assembled using Bi@NSC as the anode and K_*x*_Mn[Fe(CN)_6_] as the cathode delivered an energy density of 295 W h kg^−1^ with excellent stability for 800 cycles and 83% capacity retention. Intermetallic materials hold the advantage of high capacity contributed by both metallic components. Intermetallic Sn–Sb has been proven to have a positive impact on the durability of KIBs when compared to pure Sn.^77^ Although a carbon matrix can accommodate volume expansion, it decreases the volumetric energy density of the hybrid and causes a low ICE due to its large surface area.^78^ Tuan *et al.* reported an interesting strategy of hybridising Bi_*x*_Sb_1−*x*_ nanocrystals with a P matrix (Bi_*x*_Sb_1−*x*_@P). Both Bi and Sb have a large volume expansion when alloying with K and forming an intermetallic compound can reduce the volume expansion due to the lattice softening effect of Sb, subsequently minimising electrode pulverisation. Moreover, P as the matrix can support and control the growth of Bi_*x*_Sb_1−*x*_ nanocrystals and buffer the volume expansion, further improving the K kinetics and electronic conductivity. Bi_0.5_Sb_0.5_ prepared by a solution precipitation method showed a small diameter of 6.35 nm was embedded in an amorphous P matrix that had a mass loading of ∼9.59 wt% (Fig. 10d). The hybrid nanostructure exhibited an excellent performance of 258.5 mA h g^−1^ at 6.5 A g^−1^ and durability of 1000 cycles (Fig. 10e). A control sample of Bi_0.5_Sb_0.5_@P was prepared by ball milling but showed a rapid capacity decay over 280 cycles (Fig. 10f).

### 3.6 Organic hybrid nanostructures

**Fig. 11:**
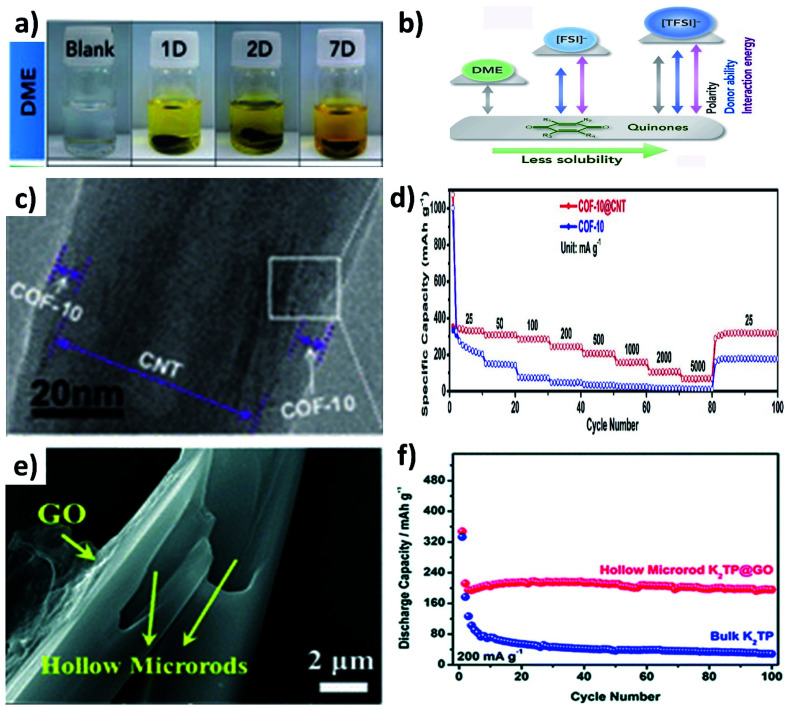
(a) Digital photographs of the dissolution of Calix[4]quinone in DME and (b) schematic comparison of polarity, donor ability, and quinone-solvent interactive energy between DME and ionic liquids. Reproduced with permission. Copyright 2019, Elsevier. (c) TEM image of COF@CNT and (d) rate performance of the COF and COF@CNT. Reproduced with permission. Copyright 2019, American Chemical Society. (e) SEM image of K_2_TP@GO and (f) cycling stability of bulk K_2_TP and K_2_TP@GO hybrid structures. Reproduced with permission. Copyright 2018, Royal Society of Chemistry.

Organic materials are made of light elements (*e.g.*, C, H, N, O and S), which can provide abundant active sites to enable the electrochemical K storage reaction and low energy barriers to facilitate K diffusion. However, there are limiting factors such as high solubility in electrolytes and poor electronic and ionic conductivity, which significantly hinder the use of organic materials in electrochemical K storage.^79–81^ For instance, potassium 1,1-biphenyl-4,4-dicarboxylate (K_2_BPDC) and potassium 4,4-*E*-stilbenedicarboxylate (K_2_SBDC) exhibited capacity fading even at a low current density due to their solubility in the electrolyte of 1 M potassium bis(fluorosulfonyl)amide (KFSI) in ethylene carbonate and dimethyl carbonate (EC : DMC).^79^ The dissolution of organic materials in electrolytes can be visually observed from the colour change of the electrolyte, as seen from the dissolution of Calix[4]quinone turning the colourless dimethoxyethane (DME) solvent to yellow over a period of time (Fig. 11a), due to the strong polarity between Calix[4]quinone and DME (Fig. 11b).^82^ In this section, we will discuss the utilisation of hybrid nanostructures to reduce the dissolution of organic materials and the simultaneous enhancement of their electrical conductivity.

The widely used hybrids of inorganic materials contain carbon materials such as graphene,^79^ multiwalled CNTs,^83,84^ graphene nanotubes^85^ and super P.^86,87^ As an example, the hybrid of a covalent organic framework (COF) and CNTs (COF@CNT) was reported to facilitate electronic and K^+^ transport.^88^ COF@CNT was prepared by a solvothermal method and provided a uniform coating of the COF on the surface of CNTs (Fig. 11c). Due to the hybridisation with CNTs, the rate performance of COF@CNT at 5 A g^−1^ was enhanced by ∼5 times when compared to the pristine COF (Fig. 11d). Using a similar approach, Chen's group hybridised potassium terephthalate (K_2_TP) with CNTs and demonstrated the performance of full cell KIBs.^84^ The full cell was assembled from the K_2_TP@CNT anode and potassium iron hexacyanoferrate (KFeHCF) cathode and delivered a reversible specific capacity of 110 mA h g^−1^ with 90% capacity retention after 60 cycles. Also, the rate performance of the full cell was observed with a deliverable specific capacity of 58 mA h g^−1^ at 20C. It has been reported that hybridising K_2_TP with 2D GO can restrain the dissolution of K_2_TP during charge/discharge processes,^89^ where GO was wrapped over hollow K_2_TP microrods by freeze drying the mixture of GO and K_2_TP (Fig. 11e). The reduction of the dissolution of K_2_TP in the electrolyte was evidenced by the durability of the K_2_TP@GO electrode that retained 92.3% capacity after 100 cycles with a CE of 99%. In contrast, K_2_TP microrods retained a specific capacity of only 5.2% after 100 cycles (Fig. 11f). Furthermore, graphene nanotubes (GNTs) hold the merits of graphene and CNTs, and the vitamin K (VK)@GNOT hybrid has been demonstrated to mitigate the challenges of pristine VK anodes for KIBs.^85^

### 3.7 Metal–organic framework hybrid nanostructures

**Fig. 12:**
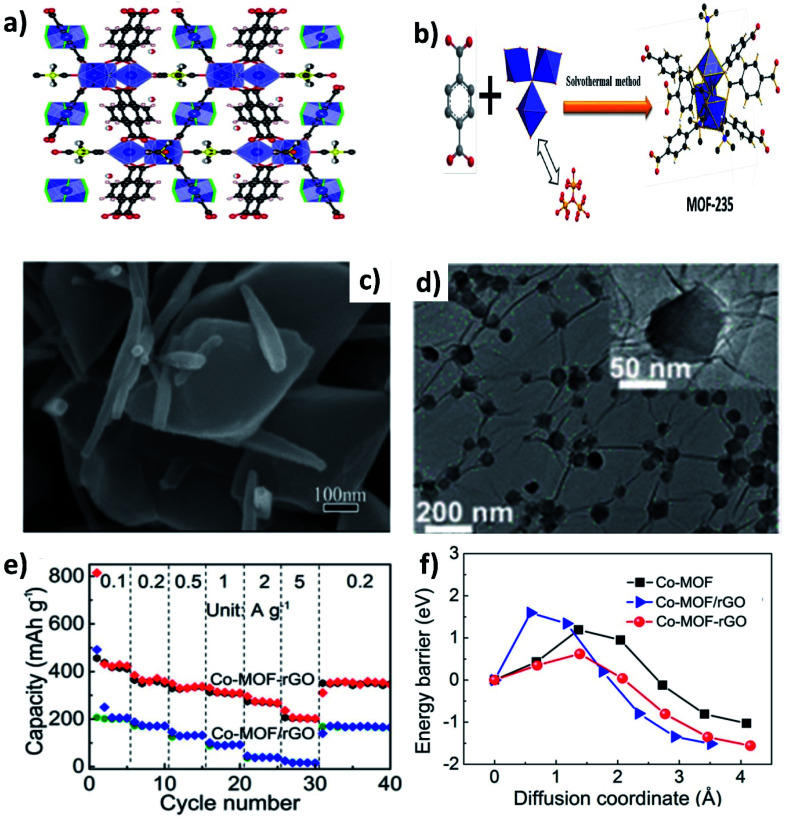
(a) Crystal structure of MOF-235 (red, black and blue spheres represent O, C and Fe, respectively), (b) schematic of the synthesis of MOF-235 by a solvothermal method, and (c) SEM image of the MOF/CNT hybrid. Reproduced with permission. Copyright 2020, Elsevier. (d) TEM image of the Co-MOF/rGO hybrid, (e) rate performance of the Co-MOF/rGO hybrid and Co-MOF–rGO mixture, and (f) energy barriers of K^+^ diffusion in the Co-MOF/rGO hybrid, Co-MOF–rGO mixture, and pristine Co-MOF. Reproduced with permission. Copyright 2020, American Chemical Society.

Metal–organic frameworks (MOFs) have gained significant interest due to their open framework structure formed by linking organic and inorganic units.^90^ The open framework facilitates reversible K^+^ insertion/extraction while the inorganic units act as redox active sites to enable multielectron transfer; hence, MOFs are considered to be potential electrode materials for KIBs. However, the rate performance of MOFs is unsatisfactory due to their poor electrical conductivity. The K storage performance of a Ti based pristine MOF (MIL-125) exhibited a specific capacity of 208 mA h g^−1^ at 10 mA g^−1^ and cycling durability can be maintained for 2000 cycles at a low current density, but the capacity significantly dropped to 56 mA h g^−1^ at 200 mA g^−1^.^91^ In this regard, hybridising MOFs with carbon materials can improve the electrochemical performance at high current densities. Deng *et al.* hybridised MOF-235 with multiwalled CNTs,^92^*via* a one-pot solvothermal method (Fig. 12a and b), obtaining a uniform coverage of CNTs over MOF-235 (Fig. 12c). The MOF@CNT hybrid exhibited a specific capacity of 101 mA h g^−1^ at 200 mA g^−1^, which was higher than that of pristine MOF-235 (<100 mA h g^−1^ at 50 mA g^−1^). Xu's group prepared uniformly distributed Co-MOF nanocrystals (∼70 nm) over rGO (Fig. 12d).^93^ The chemical interaction between Co-MOF and rGO in the hybrid structure was shown to be crucial for the observed electrochemical performance. Chemically bonded Co-MOF@rGO delivered a capacity at 5 A g^−1^ that was ∼6.7 times higher than that of a physical mixture of Co-MOF and rGO (Co-MOF–rGO, Fig. 12e). This was due to the lower energy barrier of K^+^ diffusion in the hybrid nanostructure when compared to Co-MOF–rGO and pristine Co-MOF (Fig. 12f).

## 4. Hybrid nanostructures for K–S and K–Se batteries

K–S and K–Se batteries hold promise for large-scale energy storage but their development is still in its infancy and facing great challenges, as outlined in Section 2.2. Up to now, research on K–S and K–Se batteries has been conducted around the structural design of the S/Se cathode and the electrochemical stripping/plating of the K anode. This section will focus on the advancement of the cathode design, as hybrid nanostructures are less relevant to K stripping/plating and its progress in K–S and K–Se batteries has been summarised in previous reviews.^94,95^ Due to the similar working mechanism and nature of the challenges for K–S and K–Se batteries, the two battery systems will be discussed in parallel.

### 4.1 Carbon-based hybrid nanostructures

It has been proven effective to fabricate S/Se cathode structures by hybridising with carbon in accommodating S/Se, enhancing electronic conductivity and accommodating the volume change that occurs during the conversion reaction between S/Se and K. The freedom of tuning the electronic conductivity, surface area and porosity of carbon makes it possible to design hybrid nanostructures with the focus of addressing specific issues. In Section 4.1.1, specific attention is given to the concept of controlling the porosity of carbon hosts to balance between maximum sulphur loading and inhibiting polysulphide/polyselenide dissolution. Although porous carbon can physically confine active sulphur, the use of an additional carbon component can offer extra functionalities such as enhancing the electronic conductivity and mechanical strength of the S/Se cathode and providing additional free space for S/Se loading. The extra functionalities are discussed in Section 4.1.2.

### 4.2 Single carbon hybrid nanostructures

Controlling the porosity of the carbon matrix in hybrid nanostructures aids to control the S/Se loading and shuttle effect of polysulphides/polyselenides, and an appropriate pore size is important. If the pores are too small, S/Se is less likely to be impregnated into the host, and the K diffusion is negatively affected; however, a high S/Se loading is possible if the impregnation is successful. In contrast, a large pore size could lead to a high S/Se loading and facilitates K diffusion into the pores but results in an easier dissolution of polysulphides/polyselenides, compared to a small pore size.

**Fig. 13:**
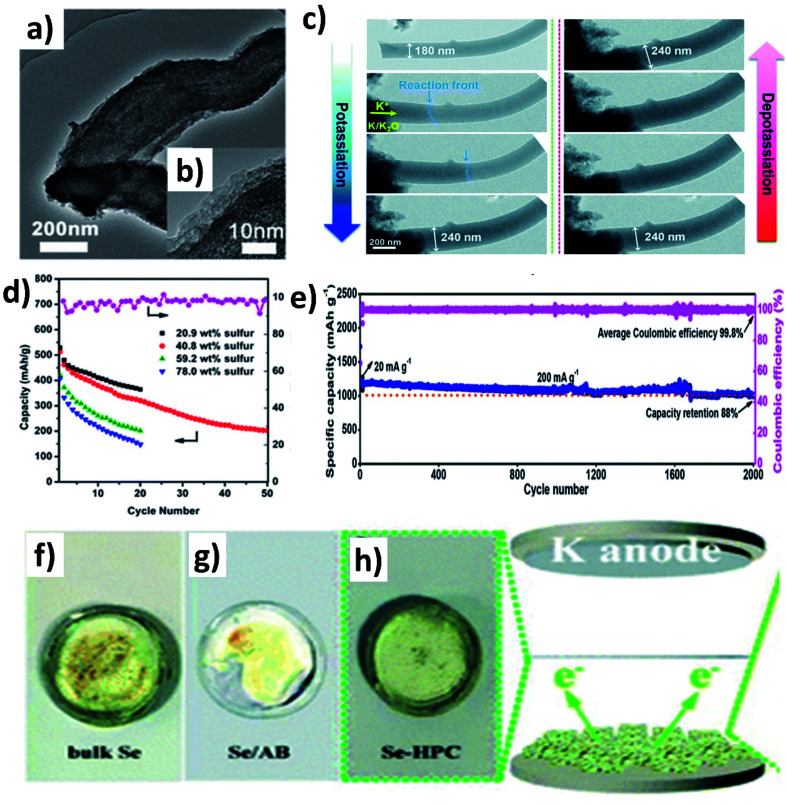
(a) TEM image of PANI@CMK/S, (b) HRTEM image (inset) showing the PANI deposited on the surface of CMK-3 and (d) cycling performance of PANI@CMK/S with different S loadings. Reproduced with permission. Copyright 2014, American Chemical Society. (c) *In situ* TEM images of PCNF/S during (de)potassiation and (e) cycling stability of PCNF/S. Reproduced with permission. Copyright 2020, Royal Society of Chemistry. (f)–(h) Photographs of the glass fibres taken from the bulk Se, Se/AB and Se@HPC battery cells. Reproduced with permission. Copyright 2019, Royal Society of Chemistry.

A S/mesoporous carbon (CMK-3) hybrid nanostructure was prepared by Wang *et al.* and despite the high S loading, a low capacity of 285 mA h g^−1^ was obtained because the mesopores cannot well control the polysulphide shuttling.^96^ Chen *et al.* modified a hybrid nanostructure by coating a layer of polyaniline (PANI) (Fig. 13a and b)^97^ and examined various S loadings in the hybrid (20.9, 40.8, 59.2 and 78 wt%).^97^ The results showed that increasing S loading decreased the surface area and pore volume of the hybrid, as more S was deposited on the surface of CMK-3 rather than into the mesopores. Consequently, the capacity decreased from 540.5 to 396.8 mA h g^−1^ (Fig. 13d). The coating of PANI significantly increased the capacity retention from 39.4 to 62.9%, suggesting that PANI simultaneously improved the structural stability and hindered the dissolution of polysulphides. Compared to mesoporous carbon hosts, microporous hosts provide a stronger physical confinement of S/Se, which could be beneficial to suppress the shuttle effect. Xu *et al.* prepared a microporous C/S hybrid nanostructure with a particle size of 500 nm, a S loading of ∼40 wt%, and a pore size less than 1 nm.^98^ Most of the S in the hybrid existed in the form of S_2_ and S_3_ rather than large S_8_ molecules. A high reversible capacity of 1198.3 mA h g^−1^ was obtained at 20 mA g^−1^, and 72.5% capacity was retained after 150 cycles. The same group further improved the durability of K–S batteries without compromising the capacity by incorporating S into one-dimensional (1D) carbon nanofibres that acted as a supporting matrix for the small S molecules.^99^ The hybrid porous nanofibre/small S (PCNF/S) nanostructure with a diameter of 150 nm kept its morphology after the uniform distribution of S (mass loading ∼25 wt%) inside of PCNF. Using the hybrid as a K–S battery cathode, the *in situ* TEM measurement (Fig. 13c) showed that the diameter of PCNF/S increased from 180 to 240 nm during the discharge process and the volume expansion was about 33%; however, no major structural change/collapse was observed. The hybrid nanostructure delivered a remarkably high capacity of 1390 mA h g^−1^ at 20 mA g^−1^ and 447 mA h g^−1^ at 4 A g^−1^. The durability of the cell was tested for long-term 2000 cycles showing a capacity retention of 88% (Fig. 13e). Hierarchically porous carbon has the dual benefit of enhancing the loading of active material and reducing the loss of active material. To this end, Huang *et al.* incorporated Se into a micro–mesoporous hollow carbon matrix (Se@HPC) by the melt diffusion technique.^100^ The co-existence of micro- and meso-pores in Se@HPC enabled a 42 wt% Se loading inside the pores rather than on the surface, which contributes to the suppression of the polyselenide dissolution. It was clearly seen by the different colour of the separators taken out of the bulk Se, Se/acetylene black (AB), and Se@HPC cells (Fig. 13f–h). Se@HPC delivered a capacity of 809 mA h g^−1^ at 0.1C with a capacity retention of 53.2% after 100 cycles at 0.2C. The same research group increased the loading of Se (60 wt%) inside the micro–mesoporous carbon by increasing the pore volume to 0.8733 cm^3^ g^−1^.^101^ The durability of the hybrid structure was retained for 300 cycles at 0.5C. The control of polyselenide dissolution due to the existence of dual pores in the hybrid structure was evident from almost 100% CE throughout the 300 cycles.

#### 4.2.1 Dual carbon hybrid nanostructures

**Fig. 14:**
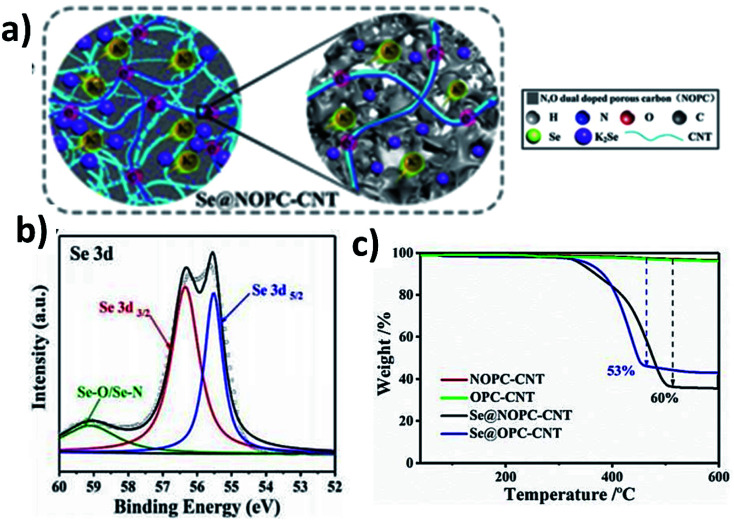
(a) Schematic illustration of the structure of CNT–ONC/Se during discharge, (b) Se 3d XPS spectra, and (c) TGA analysis of CNT-ONC/Se in a N_2_ atmosphere. Reproduced with the permission. Copyright 2018, Wiley-VCH.

Adding a second carbon component to a hybrid nanostructure can bring about extra functionality. In this regard, CNTs have been employed to form dual carbon hybrids with S/Se. The duality of CNTs intertwined into nitrogen and oxygen doped microporous carbon (CNT–ONC) allowed the improvement of the mechanical strength of the cathode and offered flexibility to the cathode. Also, the intertwined CNTs into the microporous carbon helped to fabricate a freestanding cathode without the use of a binder and current collector. Moreover, the interconnected structure of CNTs provided 3D pathways for electron transport and thus improved the reaction kinetics of the cathode (Fig. 14a). The good mechanical properties of CNTs buffered electrode volume expansion.^102^ CNTs and ONC formed an intertwined network structure, and the presence of the O and N dopants enabled a strong affinity between Se and carbon due to the formation of Se–N and Se–O bonds (Fig. 14b). The interspace between CNTs and microporous carbon provided addition space for Se loading; hence, a high amount of Se (50–60 wt%) was successfully impregnated into the carbon (Fig. 14c). The K–Se battery delivered a reversible capacity of 273 mA h g^−1^ at a rate of 5 A g^−1^ and an excellent cycling stability of 700 cycles at 0.8 A g^−1^. The study found that not only did the O and N dopants form a strong bonding with Se, but they also had a strong chemical affinity with K_2_Se, potentially contributing to reducing its dissolution and enabling long-term stability of the hybrid cathode.

### 4.3 Small S(Se) hybrid nanostructures

**Fig. 15:**
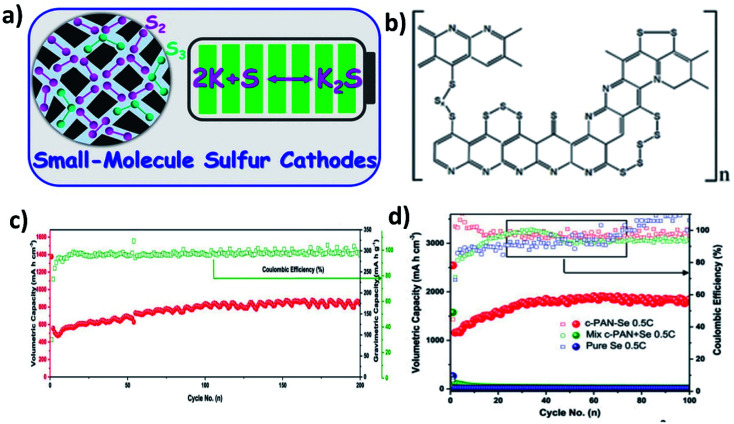
(a) Schematic illustration of the conversion of small S_2_–S_3_ molecules directly to K_2_S. Reproduced with permission. Copyright 2019, American Chemical Society. (b) Schematic of the structure of SPAN. Reproduced with permission, Copyright 2020, Wiley-VCH. (c) Cycling performance of Se_1_/PAN at a rate of 5C and (d) the comparison of the cycling performance of Se_1_/PAN, physically mixed Se + PAN and pure Se. Reproduced with permission. Copyright 2017, Elsevier.

When using large cyclo-S_8_/Se_8_ molecules in hybrid nanostructure cathodes, the molecules first convert to long chain high-order polysulphide/polyselenide products (S_*n*_^2−^/Se_*n*_^2−^, 4 < *n* < 8) before reaching the end of discharge (K_2_S/K_2_Se). The high-order chain molecules have a stronger tendency to dissolve in electrolytes than the low-order molecules (2 < *n* < 4),^95^ causing a more severe shuttle effect. Therefore, using small S/Se molecules as the starting materials in hybrid nanostructures can bypass the high-order discharge products, and even there is a trade-off with capacity (Fig. 15a). It has been found that encapsulating S into pyrolysed polyacrylonitrile (SPAN) can lead to the formation of small S molecules that were chemically bonded with the PAN backbone through C–S covalent bonds (Fig. 15b).^103,104^ As a result, the idea of using small S/Se molecules as the starting materials has been centred around the use of SPAN. Lu *et al.* applied this approach and took a step further to optimise the S mass loading in the hybrid through different annealing temperatures (350, 450 and 550 °C).^105^ Their results showed that when increasing the temperature, the surface area of the hybrid decreased and the pore volume increased, which in turn played an important role in determining the S loading in the hybrid. The pore volume of SPAN at different temperatures of 350, 450 and 550 °C are 2–5, 2–7 and 10–50 nm, respectively, giving rise to a 39.25 wt% S loading at 450 °C. This sample delivered the highest capacity and the best durability in K–S batteries among the three, suggesting that there existed a balance between the S loading, micro–mesoporous structure, and high conductivity of SPAN, and thus care needs to be taken when designing such kinds of hybrid nanostructures. Se_1_ was shown to directly form K_2_Se in a K–Se battery, without the formation of intermediate soluble polyselenides.^106^ A Se_1_/PAN hybrid nanostructure with a Se loading of 40 wt% delivered a high reversible capacity of 652 mA h g^−1^ in the first cycle and sustained the capacity for 200 cycles (Fig. 15c). The authors compared Se_1_/PAN with two control samples of pristine Se_8_ and mechanically mixed Se_8_ and PAN (Se + PAN), and they found that the capacity of the control samples dropped rapidly to zero after a few cycles (Fig. 15d). These results indicated that the small Se molecules and the strong chemical bonding between Se and PAN were both crucial to achieve a stable electrochemical performance of K–Se batteries.^106^

### 4.4 Metal-based hybrid nanostructures

**Fig. 16:**
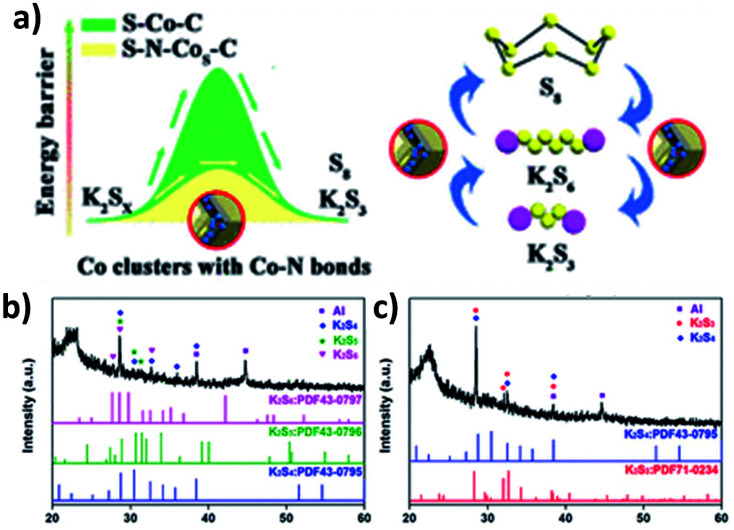
(a) Schematic illustration of the catalytic mechanism of N–Co for the polysulphide conversion reaction, and XRD analysis of S–N–Co/C discharged to (b) 1 V and (c) 0.5 V. Reproduced with permission. Copyright 2020, American Chemical Society.

As previously discussed, increasing the electronic conductivity of hybrid nanostructures and reducing the shuttle effect during battery cycles have been the main focus in the field of K–S and K–Se batteries. However, it is equally important to improve the kinetics of reversely converting polysulphides/polyselenides to S/Se, as it determines the cyclability of the batteries. Ma *et al.* introduced N-doped Co nanoparticles into a hybrid nanostructure consisting of N-doped porous carbon and S.^107^ Apart from the enhanced conductivity due to the porous carbon and the controlled S loading and volume expansion enabled by the hierarchical pores (micropores and mesopores), the catalytic ability of the N-doped Co nanoparticles allowed high reversibility of the conversion reaction. This was due to the fact that the nanoparticles enabled a low energy barrier and charge transfer resistance to promote the conversion reaction (Fig. 16a). The key to obtaining the catalytic ability was that after N-doped Co in carbon (N–Co/C) was obtained *via* the pyrolysis of ZIF-67, large Co particles were etched out using H_2_SO_4_ and so small Co particles were left in the hybrid. A heating process at 260 °C allowed the simultaneous formation of Co nanoclusters (∼3 nm) and the removal of S existing on the surface of the hybrid, as well as the homogeneous distribution of Co, N, S and C in the hybrid nanostructure. The hybrid structure S–N–Co/C with a high S loading of 62.4 wt% exhibited an initial discharge capacity of 879.4 mA h g^−1^ at 50 mA g^−1^ with a high ICE of 74.7%. This was due to the complete transition between intermediate polysulphides and K_2_S_3_ by N–Co nanoclusters. The authors carried out the XRD measurement of the electrodes discharged to 1 and 0.5 V. Intermediate polysulphides K_2_S_6_, K_2_S_5_ and K_2_S_4_ were identified (Fig. 16b) after discharging to 1 V but disappeared after discharging to 0.5 V (Fig. 16c). This confirmed the complete conversion of the intermediates to K_2_S_3_.

## 5. Summary and outlooks

Research on potassium batteries has increased rapidly in recent years, and it strongly suggests the promise of potassium batteries as next generation energy storage technology. In line with the importance of the structural design of electrodes, this review highlights hybrid nanostructures as an advantageous and versatile electrode structure for electrochemical K storage in the forms of KIBs and K–S and K–Se batteries. We outline the challenges that the three battery technologies are facing and building on this, we discuss in detail various hybrid nanostructures and their benefits in addressing the challenges and improving the electrochemical K storage performance. We emphasise that some benefits of hybrid nanostructures are universal across the three battery technologies due to their shared challenges (*e.g.*, improving charge transfer and constraining material volume change), and some benefits are crucial to a specific technology (*e.g.*, multi-functionality and catalytic ability), which once again showcases the versatility of hybrid nanostructures. Although there has been significant progress in the development of hybrid nanostructures for potassium batteries and the research activities on topic will most likely keep growing, and there still are open questions to answer and obstacles to overcome in order to further amplify the promise of hybrid nanostructures for electrochemical K storage.

### 5.1 The effect of the electrolyte

Although the use of hybrid nanostructures has shown to improve reversible capacity, durability and rate performance, the improvement of ICE has not been seen across various compositional and structural designs of the hybrids. This could be due to the large surface area of the nanostructures, particularly when carbon is involved, but could also be due to the electrolyte and binder that strongly affect the formation of a solid-electrolyte interphase (SEI) layer and irreversible side reactions. For instance, potassium salts can determine the components of the SEI layer. Potassium bis(fluorosulfonyl) imide (KFSI) tends to produce a stable SEI layer that is rich in inorganic species,^108^ but it corrodes the Al current collector on the cathode side, which imposes severa obstacles to realise the high performance obtained from a half-cell in a full-cell.^109^ The effect of electrolyte solvents on battery performance was studied in the case of graphite,^110^ but there are two or more components in a hybrid nanostructure and their interaction with the same solvent might be vastly different, which could downplay the structural benefits of the hybrid nanostructure. It is necessary to individually study the type of potassium salt, electrolyte solvent and perhaps electrolyte additive using well studied hybrid nanostructures as a model to decouple the influence of each factor.

### 5.2 Understanding the interface in hybrid nanostructures

The interface between the components is an important part of a hybrid nanostructure. However, it has been much less studied and examined compared to the hybrid as a whole. Efforts have been made to design synthetic approaches and obtain the target interface between the components, but there is little understanding of how the interface affects ion and electron diffusion as well as the interaction between the component(s) and electrolyte. This type of understanding is particularly important when there are other factors coming into play, such as interfacial defects, chemical bonding, and uneven boundaries. There is even less understanding of how the interface evolves, keeping in mind that electrochemical K storage is a dynamic process, and it is reasonable to expect that the interface would change or even restructure during long-term and repetitive charge transfer. How to record the evolution of the interface and trace the change in a micro-area remains an open question that is worth being investigated. The knowledge that will be obtained is invaluable to guide the synthesis of hybrid nanostructures.

### 5.3 Understanding the mechanism of K–S and K–Se batteries

The hybrid nanostructures of S/Se and carbon are the dominating cathodes applied in K–S and K–Se batteries. Although there have been a few studies looking at the correlation of the hybrid structure with S/Se loading and catalytic conversion of polysulphides/polyselenides, more effort needs to be devoted to these topics. The practical requirement for the S/Se content in a hybrid structure should be 65% in weight and/or 2 mg cm^−2^ in areal mass loading,^111^ but the best performance of K–S and K–Se batteries obtained so far was with a lower loading (<40 wt%) in the hybrid nanostructure. A high S/Se content might inevitably lead to a high possibility of the shuttle effect, but the exact intermediate phase transition of K_2_S_*n*_/K_2_Se_*n*_ has not yet been established, which could be complicated by the compositional and structural diversity of hybrid nanostructures. Another factor that needs further attention is the conversion reaction kinetics of intermediates. The reported K–S battery capacity (<800 mA h g^−1^) so far is less than half of the theoretical value based on the S cathode (1672 mA h g^−1^), suggesting that the multi-step phase transition might not be fully reversed, as seen in the case where K_2_S and K_2_S_2_ were observed to be the dead S species when the reversible conversion only occurred with K_2_S_3_.^112^ In this regard, electrocatalytic reversible conversion of K_2_S has started gaining attention, because the catalyst in the hybrid cathode can drive the reversible conversion of the by-products and increase the binding sites for polysulphides. This could also be appliable to K–Se batteries.

### 5.4 *In situ* studies for hybrid materials

Understanding the material formation of hybrid nanostructures is important to formulate synthesis protocols with optimised parameters. This also applies to tuning the electrochemical properties of hybrid nanostructures. Formulating synthesis protocols requires several trial-and-error experiments, which could be time-consuming. *In situ* studies during a synthesis process give information of the fundamental mechanism occurring in a synthesis process without carrying out multiple iterations in the process. For instance, *in situ* powder diffraction studies of ZnO nanoparticles during a hydrothermal process revealed the synthesis conditions to control the morphology and crystallite size of ZnO.^113^ It is worth paying attention to the implementation of techniques such as *in situ* powder diffraction, neutron diffraction, and spectroscopic measurements, as well as the combinations of these techniques during the synthesis process of hybrid nanostructures. The implementation can provide insights into the crystallisation rate, intermediate phase/precursor complexes, and reaction mechanisms of hybrid nanostructures. Besides the understanding of formation mechanism, the understanding of the electrochemical mechanism of K storage in hybrid nanostructures is equally essential. Although the macroscopic chemistry of battery reactions can be provided by *in situ* XRD and Raman studies, structural and morphological changes at the interface within hybrid nanostructures have not been fully understood during (de)potassiation. Advanced characterisation tools such as atomic-resolution scanning transmission electron microscopy (STEM) combined with electron energy-loss spectroscopy (EELS) can extract the information of the change occurring at the interface of atomic structures. Moreover, the morphological change at the interface during an electrochemical process can be visualised using the *in situ* TEM technique. Particularly, cryogenic TEM (cryo-TEM) has received tremendous attention and could be effective to preserve the interface in hybrid nanostructures.

### 5.5 Hybrid nanostructure for K–O_2_ batteries

K–O_2_ batteries are not covered in the discussion of this review because hybrid nanostructures have not been looked at in this area. A major issue responsible for the failure of K–O_2_ batteries is the growth of K dendrites caused by undesirable side reactions with the electrolyte. The protection of the anode by forming an artificial SEI layer *via* coating inorganic materials can prevent the formation of dendrites. Inorganic materials such as Al_2_O_3_ and SiO_2_ as an artificial SEI could be brittle during repeated electrochemical stripping and plating processes, so the hybrid structures of organic and inorganic components might present a surprisingly interesting opportunity to tackle the dendrite issue. The “softer” hybrid artificial SEI layer could provide flexibility to accommodate volume change and serve as a uniform passivation layer. This could enable a crack-free SEI layer and ensure a long-term cyclability. Also, K–O_2_ batteries suffer from the blocking of O_2_ diffusion at the carbon cathode because the deposited discharge product could fully cover the surface of the carbon even though it is porous. Hybrid carbons with a dual porosity or even hierarchical pores might bring about a wide range of pore sizes to selectively direct O_2_ diffusion and thus the deposition of discharge products.

## Author contributions

Conceptualisation: A. P. V. S., B. X. L. and Y. X.; writing – original draft: A. P. V. S.; writing – review & editing: A. P. V. S., B. X. L. and Y. X.; supervision and funding acquisition: Y. X.

## Conflicts of interest

The authors declare no competing interests.

## Supplementary Material
